# Associations between shoot-level water relations and photosynthetic responses to water and light in 12 moss species

**DOI:** 10.1093/aobpla/ply034

**Published:** 2018-05-24

**Authors:** Zhe Wang, Maaike Y Bader

**Affiliations:** 1 College of Life and Environmental Sciences, Shanghai Normal University, Shanghai, China; 2 CAS Key Laboratory of Mountain Ecological Restoration and Bioresource Utilization & Ecological Restoration and Biodiversity Conservation Key Laboratory of Sichuan Province, Chengdu Institute of Biology, Chinese Academy of Sciences, Chengdu, China; 3 Ecological Plant Geography, Faculty of Geography, University of Marburg, Marburg, Germany

**Keywords:** Bryophytes, gas exchange, poikilohydric, Polytrichaceae, *Sphagnum*, trade-offs, water and carbon economics

## Abstract

In vascular plants, there is a clear coupling between traits related to water and traits related to carbon economics. For bryophytes this coupling has been little studied but is expected to be strong, because in these poikilohydric plants photosynthesis varies strongly with water availability. We hypothesized that there is a trade-off between water-holding and photosynthetic capacities for mosses, resulting in a limited spectrum of possible trait combinations. At one end of this spectrum, mosses would tend to stay wet and active for long periods but would have slow photosynthetic rates. At the other end, mosses would avoid external water and dry out quicker but would have high photosynthetic capacities. We determined the water relations (water-holding and -retention capacities), photosynthetic water- and light-response curves of shoots of 12 moss species and explored the associations between these traits and their distributions among the studied species. The results partly support our hypotheses, in that the water-holding and water-retention capacities of mosses are positively related to each other and to the value and width of the optimal water-content range for photosynthesis. However, the photosynthetic capacities were specific to taxonomic groups, and the relationships between the water relations and the photosynthetic capacity are weak or inconsistent and depend strongly on the species used for analysis. The positive relationships between water-holding, water-retention and photosynthetic water-use capacities suggest two contrasting adaptations to avoid damage during dehydration: taking more time to ‘prepare’ or quick photosynthetic adjustment. However, the spectrum we hypothesized cannot be generalized for all mosses and defining a broader spectrum will require the extension of this study to a much larger number of species and including stand-level measurements of water loss and photosynthesis.

## Teaser

In vascular plants, water and carbon economics are coupled. We expected a strong coupling also in mosses, where photosynthesis depends heavily on water content: it is reduced when mosses dry out, but also when they are too wet. We hypothesized that two strategies prevail among mosses: keeping wet with slow photosynthesis, or avoiding external water, allowing short but fast photosynthesis. Water relations and photosynthetic responses to water content were indeed related to each other, but not to photosynthetic capacities. Further tests of these and other trait relationships in mosses promise an exciting new view on plant strategies for coordinating water and carbon relations.

## Nomenclature

(Ignatov and Ignatova 2003; Ignatov and Ignatova 2004; Smith 2004).

## Introduction

Plant–water relations, including water uptake, transport and loss, are important traits to describe the functioning of communities and ecosystems and have become useful predictors of the effects of global climate change on plants (Passioura 2001; Sack *et al.* 2016). Theoretically, to optimize the use of the limited resources in the environment, the acquisition of key resources such as carbon, nutrients and water of plants should be coupled (Reich 2014). There has been growing interest in the relationships between water relations and photosynthetic traits of vascular plants, revealing significant relationships between leaf hydraulic traits (e.g. hydraulic conductance) and maximum photosynthetic rates (*A*_max_) (Clearwater and Meinzer 2001; Santiago *et al.* 2004; Brodribb *et al.* 2005; Brodribb *et al.* 2007; Jones *et al.* 2010; Scoffoni *et al.* 2016; Heidi 2017). However, to date, for mosses these relationships have only been studied explicitly in two comparisons between pairs of peat mosses (Titus and Wagner 1984; Maseyk *et al.* 1999). Mosses are poikilohydric plants and their photosynthesis is highly dependent on water availability, but strategies for adapting photosynthesis to water surpluses and shortages differ strongly between species (Proctor 2001). A better understanding of the relationships between water relations and photosynthetic traits across a wide range of moss species would provide important insight into their unique ecophysiological adaptations and thereby also into the general principles of plant strategies for coordinating carbon and water relations (Glime 2017; Wang *et al.* 2017).

Being non-vascular plants and incapable to use stomata to regulate evaporation rates (in mosses, stomata are only found in the sporophytes, while the gametophytes are the dominant vegetative structures, conducting most photosynthesis), the water retention and water loss of mosses depend on plant morphology and environmental moisture conditions (Glime 2017). Water availability is the most crucial factor that controls the growth and distribution of terrestrial mosses (Birse 1958; Titus *et al.* 1983; Titus and Wagner 1984; Sonesson *et al.* 2002; Asada *et al.* 2003; Goetz and Price 2016). The water-holding capacity can be expressed by the maximum water content, WC_max_, with records from 108 to 2070 % of dry weight (Proctor *et al.* 1998), or even up to ~4000 % of dry weight for some *Sphagnum* species (Rice and Giles 1996; Maseyk *et al.* 1999). Water-retention abilities, i.e. the time needed for drying out, of different moss species also vary greatly, with exhibited ranges varying with the water availability of the habitat (Hernández *et al.* 1999; Hedenäs 2001). How these two traits, water-holding and water-retention capacity, are mutually related is not well known. However, getting to know the relationships of these traits across moss species is important to better understand their water-use strategies and to evaluate their water-cycle functions (e.g. by intercepting precipitation and reducing soil evaporation) in ecosystems (Glime 2017).

Moss photosynthesis is strongly reduced as the plants dry out, but when they hold a lot of external water the high diffusion resistance of water compared to air compromises CO_2_ exchange, so that photosynthesis is also reduced (Green and Lange 1995; Proctor 2001; Green *et al.* 2011). Because both a lack of water and excess water reduce photosynthesis, the photosynthetic response of mosses to their water content is a humpbacked curve with an optimum at intermediate water contents. Both the position (photosynthetic optimal water content, WC_opt_) and range (e.g. the 90 % range, WC_90 % range_) of the optimum are important photosynthetic water-response traits. The former indicates the optimal water content for moss photosynthesis, while the latter shows across what range of water contents the moss can maintain its photosynthesis at a relatively high level, which is important for productivity. Previous studies have found that the WC_opt_ and photosynthetic optimal water-content ranges for mosses vary from 170 to 2000 % of the moss dry weight (Dilks and Proctor 1979; Silvola and Aaltonen 1984; Alpert and Oechel 1987; Silvola 1991; Maseyk *et al.* 1999; Wagner *et al.* 2013). Thereby almost all of the values above 1000 % are for peat mosses (*Sphagnum* spp.), with the most extreme high value coming from *S. cristatum* (Glime 2017). Comparing two co-occurring *Sphagnum* species it was shown that *S. fallax* had the lowest WC_opt_ and also the lowest WC_max_ and weakest water retention (i.e. fastest drying) compared to *S. nemoreum* (Titus and Wagner 1984). Although intuitive, the suggested trade-off between maximizing water retention and maintaining photosynthesis at low water contents cannot be concluded to exist based on only two species and has not been tested across species. Apart from this example, the relationships between moss photosynthetic water-response traits and water relations, in particular the WC_max_, have not been quantified.

Apart from affecting the instant photosynthetic rates through the water content, water relations are probably also related the maximum photosynthetic rates (*A*_max_), because architectural structures affecting water exchange may also affect carbon exchange. The water content of a moss involves apoplastic, symplastic and external capillary water (Dilks and Proctor 1979; Proctor 2001). A moss species may have a high WC_max_ because it has: (i) thicker cell walls and more intra- and intercellular space (such as specialized hyaline cells and water-conducting tissues), or/and (ii) morphological structures that can hold more external capillary water. The first factor will reduce the relative photosynthetic cell volume and chloroplast number per dry weight, while the second factor is more likely to cause a CO_2_-diffusion limitation at the gas-exchange surface. Both factors will constrain the *A*_max_ (Rice 1995). On the other hand, species with weaker water-retention abilities may need a higher *A*_max_ to be productive in the short optimal period for photosynthesis. A comparison within the species *S. cristatum* showed that, indeed, the lower WC_opt_ in the green form was accompanied by a higher *A*_max_, compared to the brown form (Maseyk *et al.* 1999). In contrast, in the comparison of two *Sphagnum* species by Titus and Wagner (1984), no difference in the *A*_max_ was found. However, to the best of our knowledge, these potential trade-offs between water relations and photosynthetic capacities have never been explicitly studied in other mosses.

Water loss rates may be linked to photosynthesis in yet other ways. Water loss can be reduced by strong capillary adhesion (e.g. in fine porous structures) or by a compact growth form (Bates 1998; Koch *et al.* 2008). A compact growth form, e.g. a cushion, reduces the evaporative surface but also causes stronger self-shading, and thus restricts photosynthesis (Rice *et al.* 2014). At the shoot scale, similar trade-offs can be envisaged between increasing capillary water retention and maximizing CO_2_ diffusion into the photosynthetic tissue (Rice *et al.* 2011). Although the mechanisms of some of these trade-offs are well understood, their effect on trait relationships, i.e. how do water-related traits relate to photosynthesis-related traits, is in need of quantification.

We hypothesized that mosses need to balance between being too wet and too dry and thereby follow two main strategies:

(1) Staying wet and photosynthetically active as long as possible. These species should be able to contain a lot of water (high WC_max_) and dry slowly (slow water loss rate), while having their photosynthetic optimum at high water contents (high WC_opt_). They are expected to have a low photosynthetic capacity (*A*_max_), because they have less relative photosynthetic cell volume and the CO_2_ supply, rather than CO_2_ fixation, will be limiting much of their active time.
(2) Avoiding external water. These species should have low WC_max_ and high water loss rates, while having their photosynthetic optimum at low water contents (low WC_opt_). As their time for optimal photosynthesis is short, they should have a high *A*_max_, to be very productive before drying out completely.

Consequently, we hypothesized that the water relations and photosynthetic light- and water-response traits of mosses should be interdependent. Therefore, we determined parameters important for water relations [WC_max_ and water loss decaying constant (DC)], and photosynthetic water- [WC_opt_ and WC_90 % range_] and light-response curves in shoots of 12 moss species, and explored the associations between these traits and their distributions among the studied species. Specifically, we aimed to investigate the following questions: (i) How does the WC_max_ of moss species relate to their drying speed (DC)? (ii) How are water relations related to the response of photosynthesis to the water content (WC_opt_ and WC_90 % range_)? (iii) How are photosynthetic light-response traits related to water relations and photosynthetic water-response traits? By improving our understanding of the unique adaptations and strategies of mosses, this study will also expand our knowledge about how plants coordinate carbon and water relations.

## Methods

### Study site, focal species, sampling and pretreatment

The studied mosses were sampled at the edge of a temperate mixed coniferous and broad-leaved forest (dominated by *Picea abies* and *Fagus sylvatica*) on Lahnberge in Marburg, central Germany (8°47′E, 50°48′N, elevation 327 m). The mean monthly temperature is ~0 °C in the coldest and 18 °C in the warmest month, and the average annual precipitation is 724 mm (https://en.climate-data.org/location/22339/). The dominant moss species at the study site are *Atrichum undulatum*, *Polytrichum formosum* and *Mnium hornum*. Including these three dominant moss species, 11 common terrestrial or semi-terrestrial (on rocks, rotten logs or in ditches) moss species were sampled (the sterile shoot and reproductive shoot of *M. hornum* were considered as two different mosses and sampled separately because of their distinct appearances, so we had 12 moss types, referred to as ‘species’ for simplicity) (Table 1; see **Supporting Information—Fig. S1**).

**Table 1.** Eleven moss species (plus one species sampled also in reproductive condition) collected from the forest edge on Lahnberge in Marburg, Germany, and used for studying relationships between water relations and photosynthetic response traits. The specimens (M01–12) were deposited at the Faculty of Geography, University of Marburg. Life forms according to Mägdefrau (1982).

Family	Species	Code	Habitat	Life form
Brachytheciaceae	*Brachythecium rutabulum*	Br	Rock	Weft
Brachytheciaceae	*Eurhynchium striatum*	Es	Wood	Weft
Brachytheciaceae	*Pseudoscleropodium purum*	Pp	Soil	Weft
Dicranaceae	*Dicranum scoparium*	Ds	Soil	Turf
Hypnaceae	*Hypnum cupressiforme*	Hc	Soil	Weft
Hypnaceae	*Hypnum pallescens*	Hp	Wood	Weft
Mniaceae	*Mnium hornum* (reproductive)	Mh-R	Soil	Turf
Mniaceae	*Mnium hornum* (sterile)	Mh-S	Soil	Turf
Polytrichaceae	*Atrichum undulatum*	Au	Soil	Turf
Polytrichaceae	*Polytrichum formosum*	Pf	Soil	Turf
Sphagnaceae	*Sphagnum auriculatum*	Sa	Soil	Turf
Sphagnaceae	*Sphagnum palustre*	Sp	Soil	Turf

Four samples of each species were obtained from separated patches (any two of the patches were >20 m apart). The mosses were collected with the underlying substrate, sealed in plastic bags and brought to the laboratory. The samples were kept in a moist and shaded environment for ~2 days. One day prior the experiments, the litter, bark and mixed-in mosses of other species were removed carefully. All of the samples were washed with distilled water to clean the dust and mud. Dead tissue was removed, and the shortest possible caulidium was retained (to not detach the phyllidia), and the green moss sections were collected as the final samples.

### Gas-exchange measurements

A portable open-flow infrared gas analyzer (GFS-3000, Walz, Effeltrich, Germany) with a moss cuvette (cuvette for Lichens/Mosses 3010-V32) was used to determine the photosynthetic light- and water-response curves of the studied species. A cold-trap device (Cold Trap KF-18/2B, Walz, Effeltrich, Germany), attached between the cuvette and the analyzer and set to the incoming humidity level, was used to reduce potential problems with cross-sensitivity between water and CO_2_ signals of the infrared gas analyzer **[see****Supporting Information—Fig. S2****]**. Cuvette conditions resembled ambient conditions with temperature set ~2 degrees warmer than ambient at 20 °C, relative humidity following ambient conditions (around 80 %), and CO_2_ concentration set to 400 ppm. Light levels were set depending on the measurement. The flow rate of the system was set at 600 µmol s^−1^, which allowed a sufficient CO_2_-exchange signal.

We first determined the water-response curves, at an estimated saturating light intensity of 400 μmol photons m^−2^ s^−1^ photosynthetically active radiation (PAR), and then used the optimal water content (WC_opt_) derived from these curves for the subsequent measurements to determine light-response curves. After submersion in distilled water for 2 min, the samples were put on a mesh for 2 min to let excess water drip off. We did not use paper towel to absorb the surface water because the external capillary water plays an important role in moss physiology (Proctor 2000; Proctor 2001) and was very relevant to our measurements. For prostrate mosses, the shoots were arranged in the cuvette while avoiding mutual covering. For turf mosses, the shoots were separated and inserted into a thin dry sponge pad (which only blocked a few ventilated holes of the cuvette but did not interrupt the air mixing) on the bottom of the cuvette, making sure that the shoots were ‘standing’ but avoiding overlap. Following 30 min of light induction under 200 μmol photons m^−2^ s^−1^ PAR, the samples were enclosed in the cuvette and carbon exchange in the light (PAR = 400 μmol photons m^−2^ s^−1^) and in the dark were recorded when steady states were reached (usually after ~3 min). After each pair of measurements, the samples were weighed (Kern EW 620-3NM precision balance, Kern & Sohn GmbH, Germany) to later determine the water content based on the fresh weight and left to dry until the next measurement, ca. 30 min later. This procedure was repeated until the weight no longer changed (Wagner *et al.* 2013).

Samples with similar sizes (for prostrate mosses) or the same number of shoots (for turf mosses) as in the photosynthetic water-response curve measurements were used to determine the photosynthetic light-response curves. We could thus estimate the water content based on the fresh weight, assuming a similar biomass. The initial water content of the samples before the measurements was slightly higher than the photosynthetic optimal water content recorded from the photosynthetic water-response curves to allow for some water loss during measurement. Based on trial experiments, 13 steps of light intensity were set: 0, 25, 50, 100, 150, 200, 250, 300, 400, 500, 600, 800 and 1000 μmol m^−2^ s^−1^ PAR. Each light level lasted for ~3 min for the assimilation rate to reach a relatively steady state. The weight of the sample was determined after the measurement to make sure that the water content of the sample was still within the optimal range.

After the gas-exchange measurements, the samples were oven-dried at 70 °C for 48 h to obtain the dry mass. WC_opt_, WC_90 % range_, light compensation and saturation points (LCP and LSP), mass-based *A*_max_ and dark respiration rate (Rd) were estimated by fitting the photosynthetic light-response and water-response curves to a non-rectangular hyperbola model (Ye 2007). Fitting coefficients *R*^2^ were all above 0.90.

### Water relations

Water loss experiments were conducted to determine water-holding capacities and water loss rates (inverse of the water-retention capacity). Sample wetting pretreatments and spatial arrangements were the same as for the photosynthetic water-response curve measurement. The wet samples were weighed to obtain the maximum fresh weight and then kept under moderately cool and moist conditions [the temperature varied between 17.1 and 18.4 °C (mean 17.7 °C) and the humidity varied between 75 and 84 % (mean 81 %) during the experiment, data logger DK320, Driesen and Kern GmbH, Germany] and allowed to dry. Sample weights were recorded every 10 min during the first 60 min and at incremental intervals (12, 15, 20, 30, 60, 120, 180, 270 min, then every 3 h up to 90 h) until constant weights were obtained for all of the species. After the measurements, samples were oven-dried at 70 °C for 48 h to determine the dry weight. Water content was calculated as:

water content=(fresh weight−dry weight)/dry weight

and WC_max_ of each sample was calculated using the maximum (initial) fresh weight.

The water loss curves were fitted using exponential decay regressions (Song *et al.* 2015):

$$\text{water content}=y+a*{\text{e}}^{\left(-\text{DC}*t\right)}$$

where *t* (min) is the evaporative time and *a*, DC (decaying constant) and *y* are coefficients. The *R*^2^ were all above 0.97.

### Data analysis

Principal component analysis (PCA) was used to explore associations between the eight measured traits (WC_max_, DC, WC_opt_, WC_90 % range_, LSP, LCP, *A*_max_ and Rd). The data were then ln-transformed to satisfy normality assumptions and Pearson correlations were determined for all pairwise combinations of traits across (i) all of the 12 moss species, (ii) 10 moss species excluding the two *Sphagnum* species because of their well-hydrated living conditions and special water-related adaptations and (iii) eight moss species also excluding the two Polytrichaceae species because they are endohydric species. All of the statistical analyses were performed in PASW Statistics 19.0 (IBM, NY, USA) and Microcal Origin 9.0 (Northampton, MA, USA). Statistical results were considered significant when *P* ≤ 0.05.

## Results

**Figure 1.**
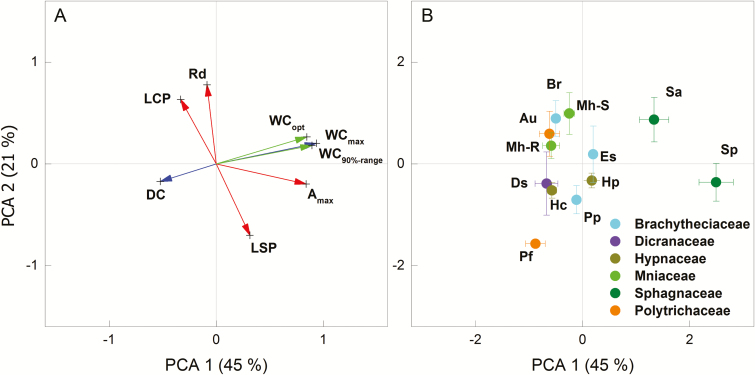
Principal component analysis of moss functional traits. (A) Loading plots of the studied functional traits. Including the maximum water content (WC_max_), water loss decaying constant (DC), photosynthetic optimal water content (WC_opt_), 90 % photosynthetic optimal water range (WC_90 % range_) Light saturation and compensation points (LSP and LCP), mass-based maximum assimilation and dark respiration rates (*A*_max_ and Rd). (B) Loading plots of the 12 moss species collected from the forest edge on Lahnberge in Marburg. The codes of species names are defined in Table 1.

**Figure 2.**
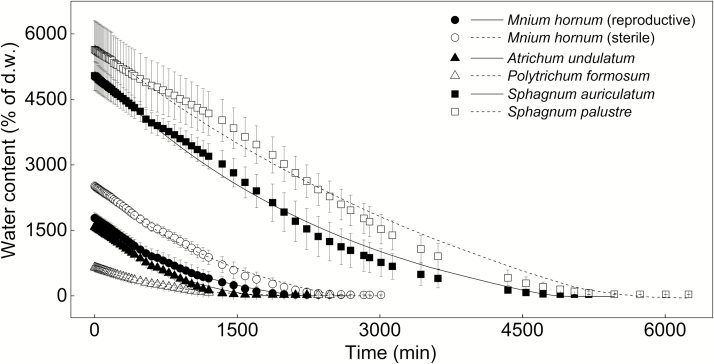
Water loss curves for six moss species collected from the forest edge on Lahnberge in Marburg. The points indicate the mean values of water content of each species (*n* = 4) over time and the error bars represent the SEs. The curves are fitted using an exponential decay regression (Song *et al.* 2015).

**Figure 3.**
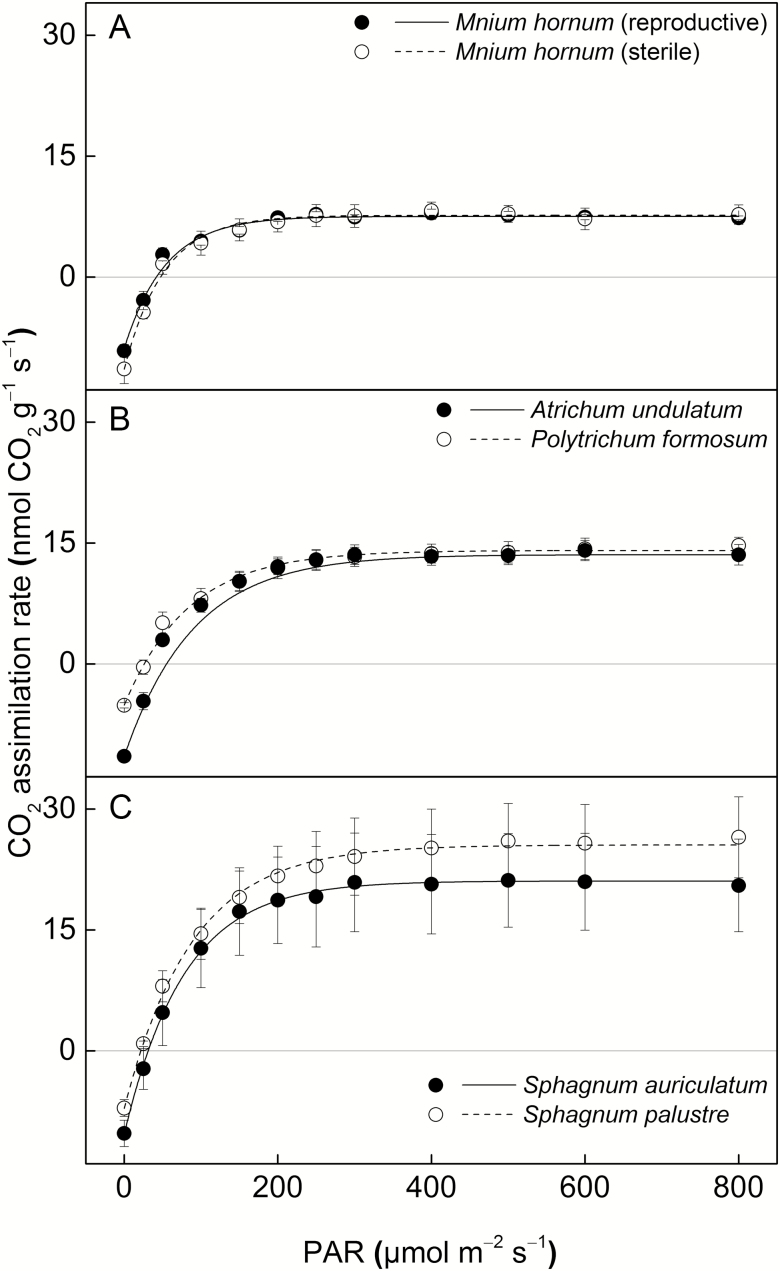
Photosynthetic light-response curves for six moss species collected from the forest edge on Lahnberge in Marburg. The points indicate the mean values of mass-based CO_2_ assimilation rates of each species (*n* = 4) at various light levels (PAR) and the error bars represent the SEs. The curves are fitted using non-rectangular hyperbola model (Ye 2007).

**Figure 4.**
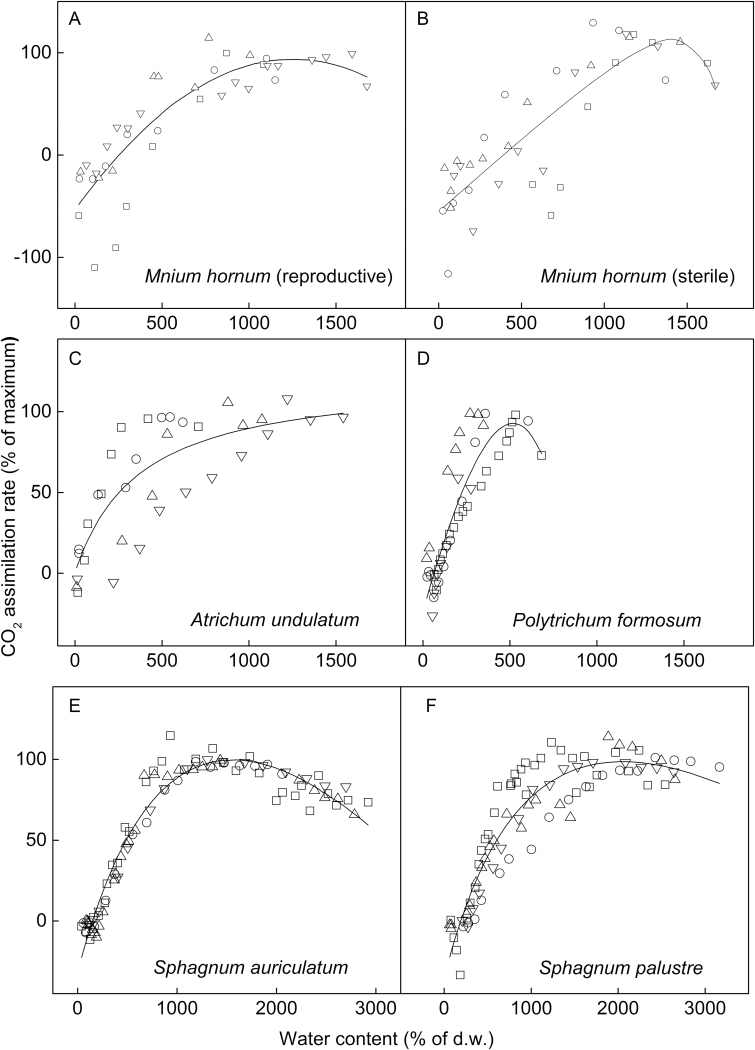
Photosynthetic water-response curves for six moss species collected from the forest edge on Lahnberge in Marburg. The points indicate the percentage of maximum assimilation rates of each replicate (*n* = 4) at various moss water contents. The curves are fitted using a non-rectangular hyperbola model (Ye 2007).

The first three PCA factors accounted for 81 % of the total variance (factor 1 explained 45 % and factor 2 explained 21 %) (Fig. 1; see **Supporting Information—Table S1**). WC_max_, DC, WC_opt_, WC_90 % range_ and *A*_max_ strongly influenced factor 1, while factor 2 mainly represented LSP, LCP and Rd (Fig. 1A). It was clear that the two *Sphagnum* species are exceptional and had high scores in PCA factor 1 (Fig. 1B), indicating their high WC_max_, WC_opt_, WC_90 % range_ and *A*_max_, and relatively low DC (second and third when ranked among species) compared to the other mosses (Table 2; Figs 2–4). At the other end of the first axis were the two Polytrichaceae species, whose WC_max_, WC_opt_ and WC_90 % range_ were relatively low compared to most other mosses, especially in *P. formosum*, and who dried fastest (highest DC), while their *A*_max_ ranked third and fourth after the two *Sphagnum* species.

**Table 2.** Moss functional trait values of 12 moss species collected from the forest edge on Lahnberge in Marburg, Germany (*n* = 4 for each species). Shown are mean values ± SD of maximum water content (WC_max_), water loss decaying constant (DC), light compensation point (LCP), light saturation point (LSP), maximum assimilation rate (*A*_max_), dark respiration rate (Rd), optimal water content (WC_opt_) and 90 % photosynthetic optimal water range (WC_90 % range_). Species codes can be found in Table 1.

	DC (*10^−3^)	WC_max_ (% of d.w.)	WC_opt_ (% of d.w.)	WC_90 % range_ (% of d.w.)	*A*_max_ (nmol CO_2_ g^−1^ s^−1^)	Rd (nmol CO_2_ g^−1^ s^−1^)	LCP (μmol photons m^−2^ s^−1^)	LSP (μmol photons m^−2^ s^−1^)
Br	0.61 ± 0.20	2271 ± 204	777 ± 163	629 ± 97	4.85 ± 1.00	8.99 ± 1.01	92.08 ± 37.29	408 ± 80
Es	0.50 ± 0.10	2388 ± 205	1172 ± 228	880 ± 281	8.84 ± 1.56	8.50 ± 3.82	58.74 ± 26.63	510 ± 80
Pp	0.54 ± 0.21	1991 ± 53	693 ± 102	648 ± 62	10.72 ± 1.24	6.20 ± 2.49	32.67 ± 5.84	503 ± 99
Ds	0.72 ± 0.43	1573 ± 195	612 ± 201	385 ± 310	6.07 ± 2.17	6.14 ± 2.05	53.15 ± 39.11	459 ± 209
Hc	0.42 ± 0.12	1999 ± 66	389 ± 65	378 ± 25	6.44 ± 3.12	5.61 ± 2.13	35.41 ± 8.82	412 ± 131
Hp	0.24 ± 0.09	2386 ± 375	829 ± 53	668 ± 122	13.97 ± 1.94	7.22 ± 1.08	29.42 ± 5.37	445 ± 39
Mh-R	1.08 ± 0.63	1782 ± 241	1092 ± 272	336 ± 129	7.88 ± 0.41	9.42 ± 1.67	36.45 ± 12.05	301 ± 19
Mh-S	0.71 ± 0.27	2512 ± 204	1218 ± 206	458 ± 57	8.14 ± 2.45	11.57 ± 3.63	48.53 ± 15.87	304 ± 78
Au	1.25 ± 0.27	1581 ± 145	861 ± 418	344 ± 161	13.98 ± 2.37	15.00 ± 6.18	39.14 ± 7.02	384 ± 81
Pf	1.49 ± 0.35	652 ± 103	576 ± 207	156 ± 102	14.47 ± 2.73	5.47 ± 0.65	26.44 ± 4.19	547 ± 63
Sa	0.36 ± 0.25	5041 ± 662	1516 ± 135	1128 ± 122	21.83 ± 11.95	10.94 ± 3.15	37.72 ± 33.45	387 ± 19
Sp	0.28 ± 0.10	5636 ± 1335	2056 ± 498	1447 ± 102	35.55 ± 11.48	6.85 ± 2.04	19.77 ± 5.12	575 ± 109

**Figure 5.**
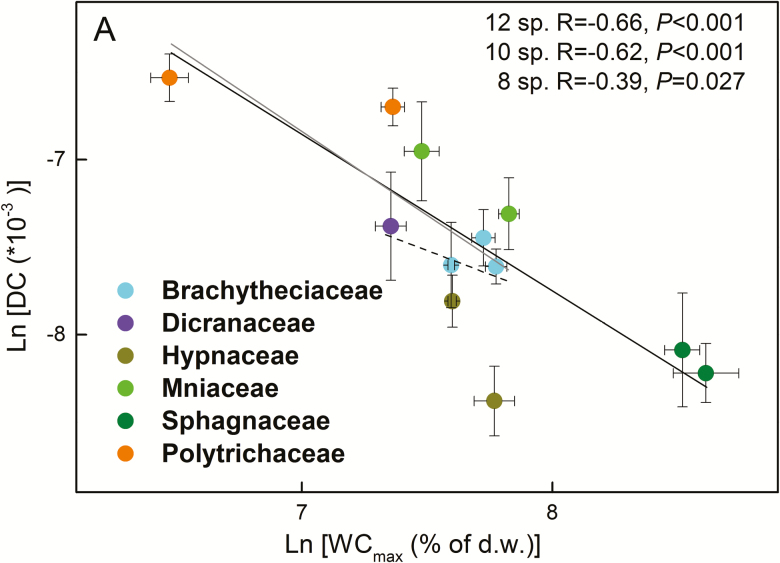
Pearson correlations between maximum water content (WC_max_) and water loss decaying constant (DC) (ln-transformed) of 12 moss species collected in Marburg. The points indicate the mean values of each species (*n* = 4) and the error bars represent the SEs. Solid, grey and dashed lines indicate the significant trait relationships when 12, 10 (the two *Sphagnum* species excluded) or 8 species (both *Sphagnum* and Polytrichaceae species excluded) were used for the analysis, respectively. Statistical results were considered significant when *P* ≤ 0.05.

**Figure 6.**
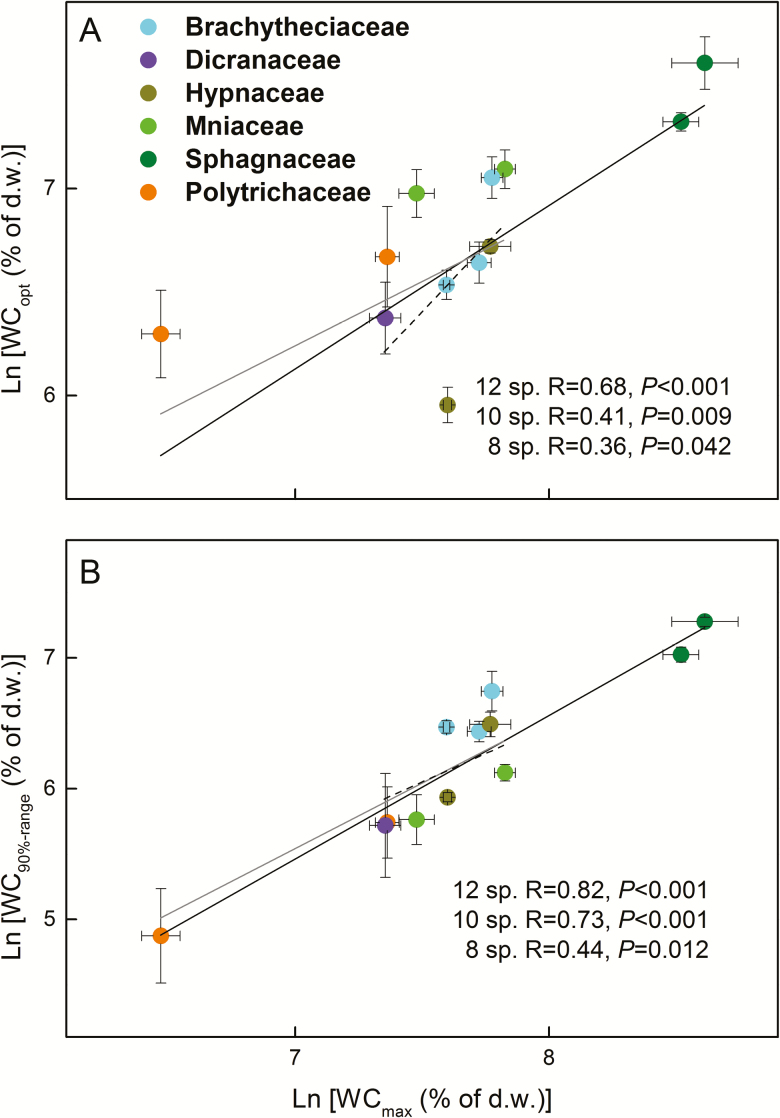
Pearson correlations between maximum water content (WC_max_) and photosynthetic water-response traits (ln-transformed) of 12 moss species collected in Marburg: (A) WC_max_ and photosynthetically optimal water content (WC_opt_); (B) WC_max_ and 90 % photosynthetically optimal water range (WC_90 % range_). The points indicate the mean values of each species (*n* = 4) and the error bars represent the SEs. Solid, grey and dashed lines indicate the significant trait relationships when 12, 10 (the two *Sphagnum* species excluded) or 8 species (both *Sphagnum* and Polytrichaceae species excluded) were used for the analysis, respectively. Statistical results were considered significant when *P* ≤ 0.05.

**Figure 7.**
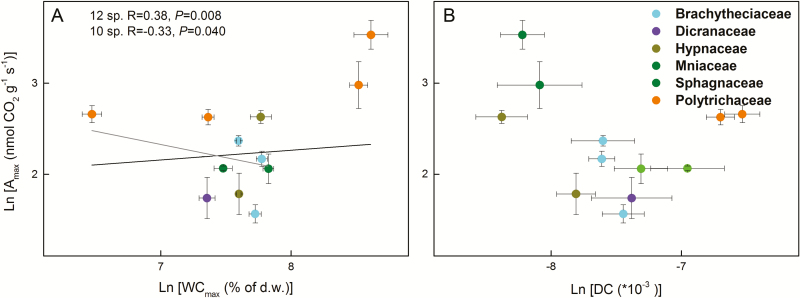
Pearson correlations between water relations and maximum assimilation rates (*A*_max_) (ln-transformed) of 12 moss species collected in Marburg. (A) Maximum water content (WC_max_) and *A*_max_; (B) water loss decaying constant (DC) and *A*_max_. Solid, grey and dashed lines indicate the significant trait relationships when 12, 10 (the two *Sphagnum* species excluded) or 8 species (both *Sphagnum* and Polytrichaceae species excluded) were used for the analysis, respectively. Statistical results were considered significant when *P* ≤ 0.05.

**Figure 8.**
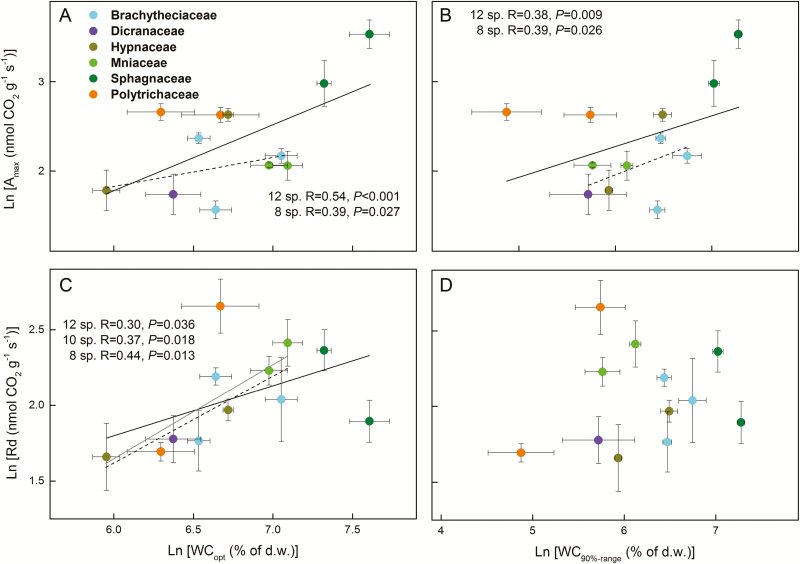
Pearson correlations between photosynthetic water-response traits and metabolic rates (ln-transformed) of 12 moss species collected in Marburg. (A) Photosynthetically optimal water content (WC_opt_) and maximum assimilation rates (*A*_max_); (B) 90 % photosynthetically optimal water range (WC_90 % range_) and *A*_max_; (C) WC_opt_ and dark respiration rates (Rd); (D) WC_90 % range_ and Rd. Solid, grey and dashed lines indicate the significant trait relationships when 12, 10 (the two *Sphagnum* species excluded) or 8 species (both *Sphagnum* and Polytrichaceae species excluded) were used for the analysis, respectively. Statistical results were considered significant when *P* ≤ 0.05.

Independent of the inclusion of the *Sphagnum* and Polytrichaceae, so whether including 12, 10 or 8 moss species in the analyses, WC_max_ was negatively related to DC (Fig. 5; see **Supporting Information—Tables S2**–**S4**). Moreover, WC_max_ was positively related to WC_opt_ and WC_90 % range_ (Fig. 6; see **Supporting Information—Tables S2****–****S4**), and DC was negatively related to WC_90 % range_ when considering all species **[see****Supporting Information—Table S2****]** or when excluding only *Sphagnum***[see****Supporting Information—Table S3****]**. When all of the species were considered in the analysis, *A*_max_ was positively related to WC_max_, WC_opt_ and WC_90 % range_, but not to DC (Figs 7 and 8; see **Supporting Information—Table S2**). However, these relationships reversed (WC_max_) or disappeared (WC_opt_ and WC_90 % range_) when the two *Sphagnum* species were excluded **[see****Supporting Information—Table S3****]**, whereas when both *Sphagnum* and Polytrichaceae species were excluded, the relationships disappeared (WC_max_) or reappeared (WC_opt_ and WC_90 % range_) **[see****Supporting Information—Table S4****]**. Dark respiration (Rd) was also related to WC_opt_, but not to the other water-related traits (see **Supporting Information—Tables S2****–****S4**; Fig. 8C), while WC_opt_ did not relate to either LCP or LSP.

## Discussion

The results partly support our hypotheses, in that the water-holding capacity (WC_max_) and water-retention capacity (DC) were positively related to each other and to the photosynthetic water-response traits. However, the relationships between the water traits and the photosynthetic capacity (*A*_max_) are weak or inconsistent and depend strongly on the species used for analysis.

### Associations among water relations

The combination of a high water-retention capacity (i.e. low drying rates, low DC) and high WC_max_ indicates an adaptation strategy to stay moist as long as possible and to avoid quick desiccation. This is not only important for maintaining photosynthetic activity for long periods (Hájek and Beckett 2008), but also because species with a higher WC_max_ tend to be more sensitive to dehydration and need more time to recover (Alpert and Oechel 1987; Seel *et al.* 1992). A slower drying rate is therefore also necessary for mosses to have more time to ‘prepare’ for the upcoming dehydration by, e.g., changing the chloroplast ultrastructure, deforming mitochondria and breaking down the vacuoles into smaller vesicles to reduce desiccation damage (Oldenhof *et al.* 2006; Cruz de Carvalho *et al.* 2012; Glime 2017). It comes as no surprise that the WC_max_ values of *Sphagnum* species were the highest among the studied species, because these have large hyaline cell to store water and have a high external water-holding capacity (Dilks and Proctor 1979; Rice 1995; Rice and Giles 1996). Additionally, their low DC (slow water loss rate) can be attributed to their large canopy size and the compact multiple-layered structures, especially in the ‘heads’ in the upper parts of the stems, which result in relatively low surface area to volume ratios, reducing evaporation rates (Proctor *et al.* 2007). In contrast, the Polytrichaceae had the lowest WC_max_ and fastest water loss rates. The low WC_max_ of these species can be explained by the low external water-holding capacity (i.e. they are endohydric mosses) and water-repellent structures on the leaves serving to allow better access of CO_2_. The fast drying is surprising, as previous studies have suggested that the Polytrichaceae have several structural characteristics, such as internal water conduction, leaf movement and leaf waxes, that should minimize desiccation rates (Sarafis 1971; Clayton-Greene *et al.* 1985). However, the internal water conduction will only help avoid desiccation when rhizoids can take up water from the substrate, but not in our experimental setting. While all studied species will dry slower when in a moss canopy than in our experiment, a limitation that would be less pronounced when studying these relationships at the stand level, for the Polytrichaceae species the cutting of the shoots will have had additionally effects on the drying speed, which should be slower under natural conditions.

Water loss control strategies of mosses can be expressed at different morphological and anatomical structural levels, from the cellular to the community level (Rice and Schneider 2004; Glime 2017). However, there is still much research needed to understand the relative importance of different levels, e.g. stand-level structural characteristics such as life forms, the surface area to volume ratio or community surface roughness (Kürschner *et al.* 1999; Zotz *et al.* 2000; Rice and Schneider 2004; Zotz and Kahler 2007), within-stand arrangements of shoots, determining, e.g., the level of capillary integration (Rice 2012), and shoot- and leaf-level characteristics such as the leaf arrangement, papillae or waxes (Sarafis 1971; Clayton-Greene *et al.* 1985), in controlling water loss rates in mosses, and how these levels interact.

### Associations between water relations and photosynthetic water-response traits

Similar to previous comparative research (Silvola 1991), we found that the WC_opt_ and WC_90 % range_ of *Sphagnum* species were the highest and those of Polytrichaceae were the lowest among the studied species. The positive relationships between WC_opt_, WC_90 % range_ and WC_max_ suggest a coupling between water-holding capacities and metabolic adaptations to these potential water contents. Mathematically, a high WC_max_ is the prerequisite for a high WC_opt_ and WC_90 % range_, but it is not a guarantee: species with a high WC_max_ can still have their optimum photosynthesis at low water contents. This was the case for *Hypnum cupressiforme* (Fig. 6), but generally the water contents and photosynthetic water responses were positively correlated. Moreover, the significant relationships between water loss rates and WC_90 % range_ indicate that species that dry slowly also maintain their net photosynthetic rates at a relatively high level across a wider range of water contents, thus causing a double advantage for total photosynthetic assimilation. In contrast, the species with lower WC_max_ (e.g. the Polytrichaceae or *Dicranum scoparium*) lose water faster and have a narrower photosynthetic optimal water range. This may indicate a quick adjustment of cell photosynthetic activities to prevent dehydration damage (Tuba *et al.* 1996).

### Associations between water relations, photosynthetic water-response traits and photosynthetic light-response traits

Relationships between *A*_max_ and WC_max_ depended on the species pool, the two *Sphagnum* species having over-proportionally large effects and even reversing the results. These species, as well as *Hypnum pallescens*, do not conform to our two alternative models: they maintain high water contents but also have high assimilation rates. For *Sphagnum* it has been shown that up to 20 % extra CO_2_ can be provided by methanotrophic bacteria located in the hyaline cells (Kostka *et al.* 2016), which would increase photosynthesis in wet conditions and may partly explain the high *A*_max_. The Polytrichaceae arguably do conform to our models, exhibiting the second strategy, i.e. showing quick drying accompanied by high assimilation rates. The other species do not show significant relationships, perhaps partly due to the lower variation in trait values and to the low remaining sample size.

The high photosynthetic potential of *Sphagnum* species at high water contents may explain the high *Sphagnum* biomass production in peatlands, with variation between species allowing successful growth in wetter or drier positions within bogs (Titus and Wagner 1984). Being able to photosynthesize well at high water contents appears to come at a metabolic cost, however, as indicated by the positive relationship between WC_opt_ and dark respiration rates (Rd), which was not caused by *Sphagnum* alone (Fig. 8C). Generally, Rd was relatively high compared to *A*_max_, which is a typical pattern in mosses (Wagner *et al.* 2014). If these mosses would live under measurement conditions, this would lead to carbon starvation, but in their natural habitat microclimatic patterns can favour the balance towards carbon gain: nights are generally cooler than days, so that the actual respiration is reduced, and the CO_2_ concentration in moss canopies can be considerably higher than ambient concentrations (Tarnawski *et al.* 1994; Holtum and Winter 2001).

The fact that we found very different results when including or excluding the morphologically unusual *Sphagnum* and Polytrichaceae suggests that the trade-off between water retention and photosynthesis depends strongly on the morphological adaptations controlling water dynamics. Therefore, we must conclude that our models cannot be generalized for all mosses. However, for a thorough test of our hypotheses, we would need to test many more than 8 or 12 species and expand the analysis to other habitat types. Including a larger species pool will also make it feasible to analyse relationships between physiological properties and morphological traits (e.g. hyaline cells), which will help to understand the functional significance of such adaptations.

Drying speed was not related to photosynthetic capacity. Also, no relationships were found between the photosynthetic responses to light (LCP and LSP) and water content (WC_opt_ and WC_90 %_), which seems to suggest that the photosynthetic adaptations to water and to light of mosses evolved independently. However, it is important to note here that our measurements were done for moss shoots rather than moss canopies, explicitly avoiding self-shading. It would be valuable to repeat these analyses with moss canopies, where we expect a strong trade-off between the water retention and light capture (Bates 1998; Zotz and Kahler 2007; Glime 2017).

## Conclusions

We found that the water-holding and water-retention capacities of mosses are positively related to each other and to the value and width of the optimal water-content range for photosynthesis. Photosynthetic capacities were specific to taxonomic groups and no general relationship with water dynamics could be recognized in the 12 species studied.

Future studies on moss photosynthesis vs. water relations trade-offs should consider the following aspects: (i) The current study showed that the relationship between photosynthetic capacity and water-holding capacity of mosses is highly dependent on the species and their living environments. Detailed studies within functionally similar sets of species, as well as broad surveys including a wide variety of functional and morphological moss types should be considered. (ii) Geographical scale is crucial in ecological research and patterns measured at small scales do not necessarily hold at larger scales (Schneider 2001). A multi-scale approach is therefore recommended, including patterns within communities and between communities in different habitats. (iii) Water content is easy to measure and correlates with many metabolic functions in mosses. However, it will be worth exploring other aspects of water relations that may be harder to measure but can more directly predict specific processes like CO_2_ diffusion. (iv) Moss canopy structure, such as the size and surface roughness, has great effects on the water-retention abilities of as well as light gradients in the canopy, thus affecting photosynthesis directly and indirectly (Zotz *et al.* 2000; Rice *et al.* 2001; Rice and Schneider 2004; Rice 2012). It remains to be investigated whether and how these morphological traits may affect the trade-off among water relations and photosynthetic water- and light-response traits.

## Sources of Funding

This research was supported by the German Research Foundation (DFG, BA 3843/3-3), the National Natural Science Foundation of China (31600316) and the Sino–German Postdoc Scholarship Program (57165010) of the China Scholarship Council and the German Academic Exchange Service (DAAD).

## Contributions by the Authors

Z.W. and M.Y.B. designed the study and wrote the manuscript. Z.W. collected the data and performed the data analysis.

## Conflict of Interest

None declared.

## Supporting Information

The following additional information is available in the online version of this article—

**Figure S1.** (a) Photos of the 12 moss ‘species’ collected in Marburg, Germany. (b) Comparison of the morphology of reproductive and sterile shoots of *Mnium Hornum*.

**Figure S2.** A cold trap (Walz KF-18/2B) was connected to the GFS-3000 control unit, between the cuvette and cuvette filter.

**Table S1.** Principal component analysis of functional traits of 12 moss species collected in Marburg, Germany.

**Table S2.** Pearson correlations among moss traits. Data from 12 moss species collected from the forest in Marburg (*n* = 4 for each species).

**Table S3.** Pearson correlations among moss traits. Data from 10 moss species (excluding two *Sphagnum* species from the total data set).

**Table S4.** Pearson correlations among moss traits. Data from eight moss species (excluding two *Sphagnum* and two Polytrichaceae from the total data set).

Supplementary Material
