# Supplementary material for: Associations between shoot-level water relations and photosynthetic responses to water and light in 12 moss species
Source: AoB Plants. 2018 May 24;10(3):ply034. doi: 10.1093/aobpla/ply034 (PMC6012793; doi:10.1093/aobpla/ply034)
Supplement: Supplementary Material [file ply034_suppl_supplementary_material.docx]

## Figure S1


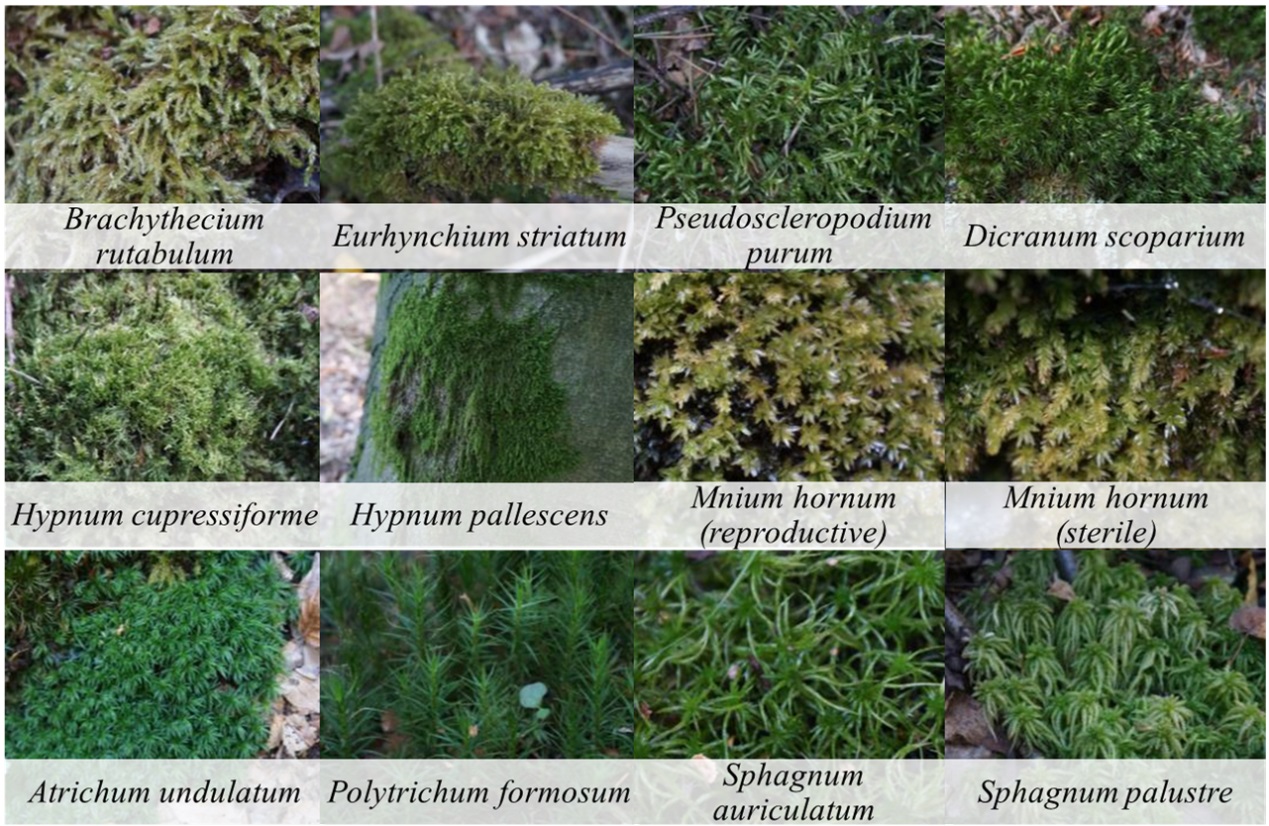


Figure S1a Photos of the twelve moss ‘species’, which actually are represented by eleven species plus two forms of *Mnium hornum*, collected in Marburg, Germany, and used to determine correlations between photosynthetic traits and water relations in mosses.


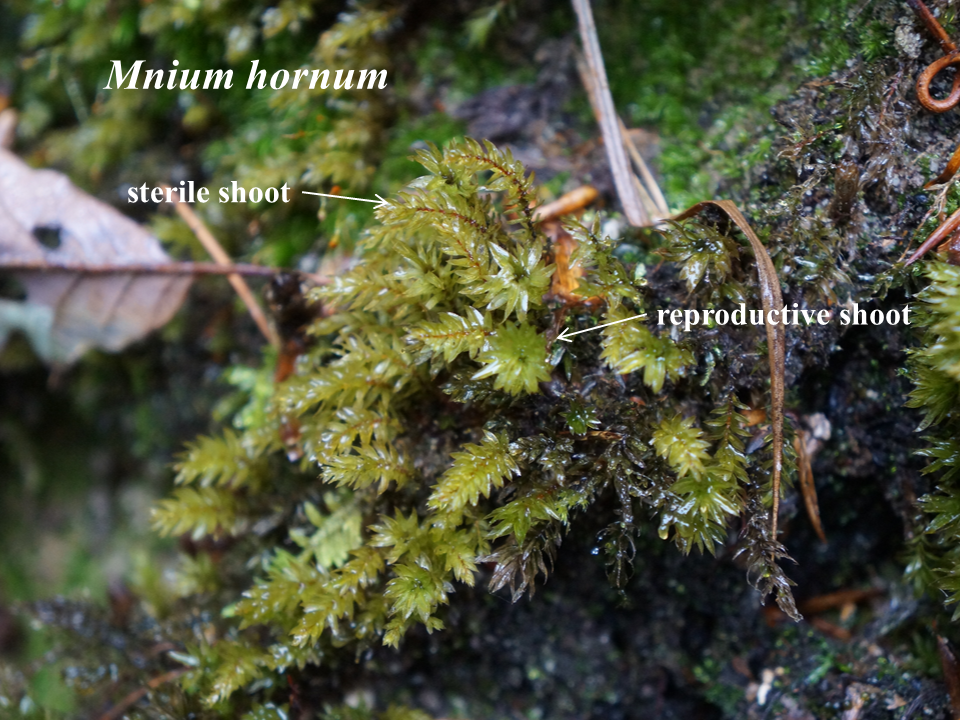
Figure S1b Comparison of the morphology of reproductive and sterile shoots of *Mnium Hornum*: the reproductive shoots are erect, with the top leaves forming rosettes, while the vegetative shoots are arcuate, with leaves spirally arranged. The trait values of the current study were similar for the two moss types, except that the sterile shoots possessed higher maximum water content than that of the reproductive shoots.

## Figure S2


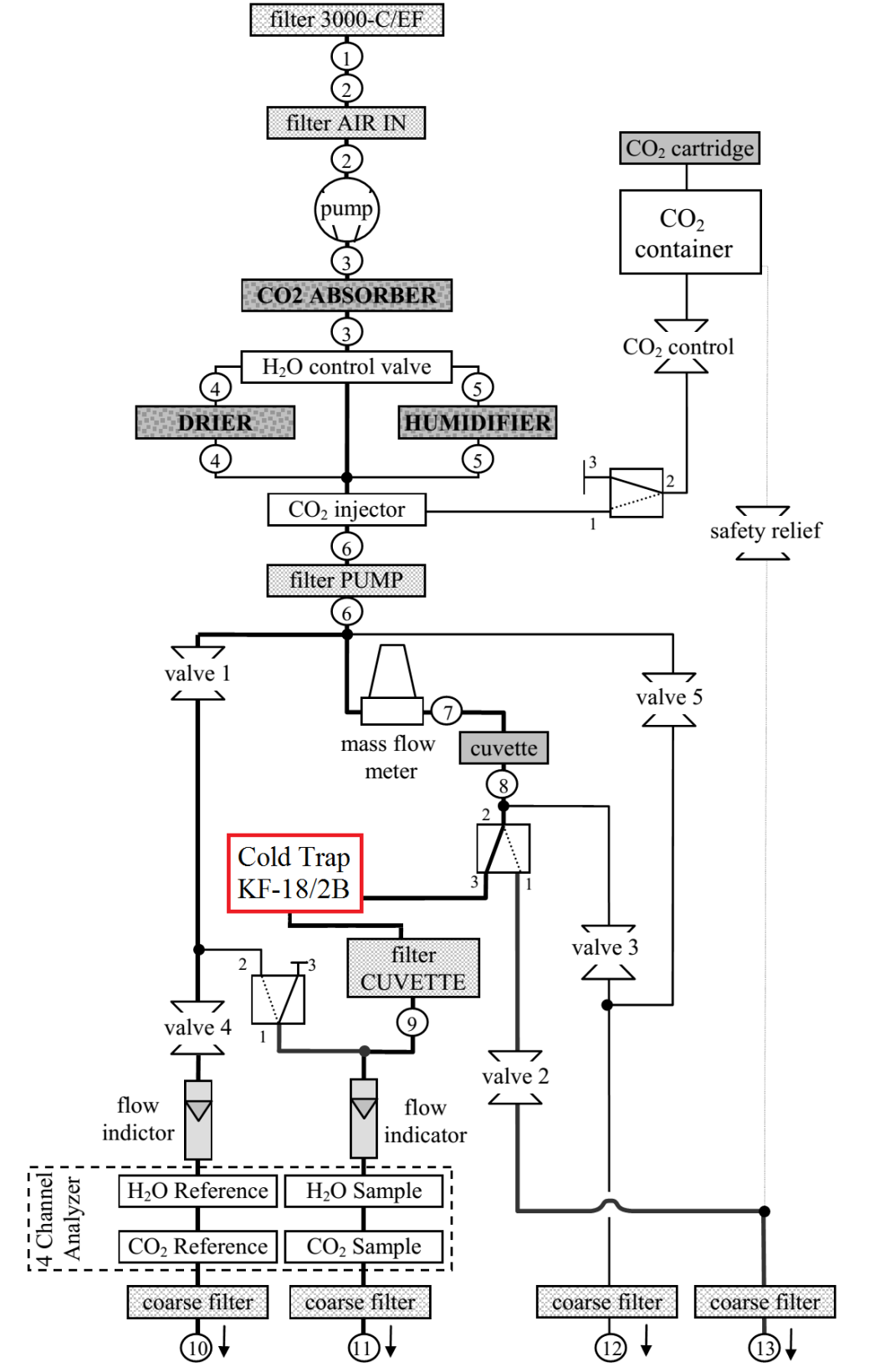


Figure S2 A cold trap (Walz KF-18/2B) was connected to the GFS–3000 control unit, between the cuvette and cuvette filter. The cooling temperature was set according to the dew point temperature of the ambient humidity, to minimize the difference in H_2_O concentration between reference and sample gas. The measuring head time lag was calibrated to account for the longer pathway of the sample gas. The diagram is modified from the pneumatic diagram of the GFS–3000 in the instruction manual by Heinz Walz GmbH.

## Table S1

Principal component analysis of functional traits of twelve moss species collected in Marburg, Germany. Shown are values of factor loadings and final communality extractions, and the percent of variance explained by each factor. Bold values indicate loadings considered valid (> 0.5) for the factor. Traits: maximum water content (WC_max_), water loss decaying constant (DC), optimal photosynthetic water content (WC_opt_), 90% optimal photosynthetic water content range (WC_90%–range_), mass–based maximum assimilation rate (A_max_), mass-based dark respiration rate (Rd), photosynthetic light compensation point (LCP) and photosynthetic light saturation point (LSP).

| Trait | Component 1 | Component 2 | Component 3 | Communality Extraction |
| --- | --- | --- | --- | --- |
| WC_max_ | **0.934** | 0.203 | -0.019 | 0.914 |
| DC | **-0.524** | **-0.173** | **0.725** | **0.830** |
| A_max_ | **0.840** | -0.198 | 0.343 | 0.863 |
| LCP | -0.333 | **0.634** | -0.497 | 0.760 |
| LSP | 0.313 | **-0.702** | -0.215 | 0.637 |
| Rd | -0.087 | **0.777** | 0.425 | 0.793 |
| WC_opt_ | **0.846** | 0.264 | 0.224 | 0.836 |
| WC_90% –range_ | **0.893** | 0.181 | -0.159 | 0.856 |
| % variance explained | 44.803 | 21.403 | 14.905 |  |

## Table S2

Pearson correlations among moss traits: maximum water content (WC_max_), water loss decaying constant (DC), optimal photosynthetic water content (WC_opt_), 90% optimal photosynthetic water content range (WC_90%–range_), mass–based maximum assimilation rate (A_max_), dark respiration rates (Rd), photosynthetic light compensation points (LCP) and photosynthetic light saturation points (LSP). Data from **twelve** moss species collected from the forest in Marburg (*n*=4 for each species).

| Trait (Ln transformed) | WC_max_ | DC | A_max_ | LCP | LSP | Rd | WC_opt_ | WC_90%–range_ |
| --- | --- | --- | --- | --- | --- | --- | --- | --- |
| WC_max_ | — |  |  |  |  |  |  |  |
| DC | -0.658^***^ | — |  |  |  |  |  |  |
| A_max_ | 0.378^**^ | -0.172 | — |  |  |  |  |  |
| LCP | -0.154 | 0.020 | -0.721^***^ | — |  |  |  |  |
| LSP | -0.001 | -0.170 | 0.427^**^ | -0.292^*^ | — |  |  |  |
| Rd | 0.169 | 0.171 | 0.023 | 0.365^*^ | -0.445^**^ | — |  |  |
| WC_opt_ | 0.681^***^ | -0.247 | 0.542^***^ | -0.222 | 0.007 | 0.304^*^ | — |  |
| WC_90%–range_ | 0.824^***^ | -0.587^***^ | 0.375^**^ | -0.102 | 0.122 | 0.058 | 0.628^***^ | — |

^***^ indicates *P* < 0.001, ^**^ indicates 0.001 ≤ *P* < 0.01 ^*^indicates 0.01 ≤ *P* ≤ 0.05.

## Table S3

Pearson correlations among moss traits: maximum water content (WC_max_), water loss decaying constant (DC), optimal photosynthetic water content (WC_opt_), 90% optimal photosynthetic water content range (WC_90%–range_), mass–based maximum assimilation rate (A_max_), dark respiration rates (Rd), photosynthetic light compensation points (LCP) and photosynthetic light saturation points (LSP). Data from **ten** moss species (excluding two *Sphagnum* species from the total dataset) collected from the forest in Marburg (*n*=4 for each species).

| Trait (Ln transformed) | WC_max_ | DC | A_max_ | LCP | LSP | Rd | WC_opt_ | WC_90%–range_ |
| --- | --- | --- | --- | --- | --- | --- | --- | --- |
| WC_max_ | — |  |  |  |  |  |  |  |
| DC | -0.620^***^ | — |  |  |  |  |  |  |
| A_max_ | -0.327^*^ | 0.171 | — |  |  |  |  |  |
| LCP | 0.321^*^ | -0.141 | -0.654^***^ | — |  |  |  |  |
| LSP | -0.230 | -0.098 | 0.396^*^ | -0.223 | — |  |  |  |
| Rd | 0.201 | 0.224 | 0.064 | 0.392^*^ | -0.423^**^ | — |  |  |
| WC_opt_ | 0.406^**^ | 0.020 | 0.180 | 0.074 | -0.179 | 0.373^*^ | — |  |
| WC_90%–range_ | 0.729^***^ | -0.484^**^ | -0.070 | 0.221 | 0.005 | 0.039 | 0.415^**^ | — |

^***^ indicates *P* < 0.001, ^**^ indicates 0.001 ≤ *P* < 0.01 ^*^indicates 0.01 ≤ *P* ≤ 0.05.

## Table S4

Pearson correlations among moss traits: maximum water content (WC_max_), water loss decaying constant (DC), optimal photosynthetic water content (WC_opt_), 90% optimal photosynthetic water content range (WC_90%–range_), mass–based maximum assimilation rate (A_max_), dark respiration rates (Rd), photosynthetic light compensation points (LCP) and photosynthetic light saturation points (LSP). Data from **eight** moss species (excluding two *Sphagnum* and two Polytrichaceae from the total dataset) collected from the forest in Marburg (*n*=4 for each species).

| Trait (Ln transformed) | WC_max_ | DC | A_max_ | LCP | LSP | Rd | WC_opt_ | WC_90%–range_ |
| --- | --- | --- | --- | --- | --- | --- | --- | --- |
| WC_max_ | — |  |  |  |  |  |  |  |
| DC | -0.390^*^ | — |  |  |  |  |  |  |
| A_max_ | 0.235 | -0.238 | — |  |  |  |  |  |
| LCP | 0.105 | 0.028 | -.642^***^ | — |  |  |  |  |
| LSP | 0.134 | -0.291 | 0.361^*^ | -0.128 | — |  |  |  |
| Rd | 0.166 | 0.230 | -0.020 | 0.439^*^ | -0.395^*^ | — |  |  |
| WC_opt_ | 0.362^*^ | 0.251 | 0.392^*^ | 0.018 | -0.103 | 0.436^*^ | — |  |
| WC_90%–range_ | 0.438^*^ | -0.140 | 0.393^*^ | 0.050 | 0.278 | 0.031 | 0.336 | — |

^***^ indicates *P* < 0.001, ^**^ indicates 0.001 ≤ *P* < 0.01 ^*^indicates 0.01 ≤ *P* ≤ 0.05.
